# Regulation of the Cardiac Sodium/Bicarbonate Cotransporter by Angiotensin II: Potential Contribution to Structural, Ionic and Electrophysiological Myocardial Remodelling

**DOI:** 10.2174/157340313805076340

**Published:** 2013-02

**Authors:** Ernesto Alejandro Aiello, Verónica Celeste De Giusti

**Affiliations:** Centro de Investigaciones Cardiovasculares, Facultad de Ciencias Médicas, Universidad Nacional de La Plata, La Plata, Argentina.

**Keywords:** Angiotensin II, calcium overload, cardiac arrhythmias, cardiac hypertrophy, electrogenic sodium/bicarbonate cotransporter, electroneutral sodium/bicarbonate cotransporter, sodium overload.

## Abstract

The sodium/ bicarbonate cotransporter (NBC) is, with the Na^+^/H^+^ exchanger (NHE), an important alkalinizing mechanism that maintains cellular intracellular pH (pHi). In the heart exists at least three isoforms of NBC, one that promotes the co-influx of 1 molecule of Na^+^ per 1molecule of HCO_3_^-^(electroneutral isoform; nNBC) and two others that generates the co-influx of 1 molecule of Na^+^ per 2 molecules of HCO_3_^-^ (electrogenic isoforms; eNBC). In addition, the eNBC generates an anionic repolarizing current that modulate the cardiac action potential (CAP), adding to such isoforms the relevance to modulate the electrophysiological function of the heart. Angiotensin II (Ang II) is one of the main hormones that regulate cardiac physiology. The alkalinizing mechanisms (NHE and NBC) are stimulated by Ang II, increasing pHi and intracellular Na^+^ concentration, which indirectly, due to the stimulation of the Na^+^/Ca^2+^ exchanger (NCX) operating in the reverse form, leads to an increase in the intracellular Ca^2+^ concentration. Interestingly, it has been shown that Ang II exhibits an opposite effect on NBC isoforms: it activates the nNBC and inhibits the eNBC. This inhibition generates a CAP prolongation, which could directly increase the intracellular Ca^2+^ concentration. The regulation of the intracellular Na^+^ and Ca^2+^ concentrations is crucial for the cardiac cellular physiology, but these ions are also involved in the development of cardiac hypertrophy and the damage produced by ischemia-reperfusion, suggesting a potential role of NBC in cardiac diseases.

## INTRODUCTION

The fine regulation of intracellular pH (pH_i_) is essential for the heart. Fluctuations of pH_i_ occur physiologically in cardiac myocytes, as during changes in heart rate [1,2], but also a major decrease can occur during pathological conditions, such as myocardial ischemia [3,4]. Four sarcolemmal ion transporters regulate pH_i_ homeostasis in order to maintain its value near to 7.2 and prevent the adverse effects of large fluctuations in pH_i_. Two of these transporters mediate acid-loading, the Cl^-^/HCO_3_^-^ exchanger (anion exchanger, AE) and the Cl^-^/OH^-^ exchanger (CHE). On the other hand, two other transporters mediate the acid-extrusion, either exporting H^+^, the Na^+^/H^+^ antiporter (NHE), or introducing HCO_3_^-^ into the cell, the Na^+^/HCO_3_^-^ symporter (NBC). 

In the present review we will specifically outline the importance of NBC in the maintenance of the cardiomyocytes pH_i_. Also, due to the relevance of Angiotensin II (Ang II) in heart function, we will review the regulation of NBC by this hormone. Finally, we will recap the knowledge about the impact of NBC on intracellular Na^+^ ([Na^+^]_i_) and Ca^2+^ concentrations ([Ca^2+^]_i_), emphasizing the potential relevance of NBC in structural and electrical cardiac disorders. 

## ROLE OF THE CARDIAC NBC IN pH_i _AND [Na^+^]_i_ REGULATION

Although the physiological role of NHE as an alkalinizing mechanism has been well demonstrated [5,6], that of NBC has long been underestimated, sometimes because the investigations were carried out in HCO_3_^-^ free-buffered solutions. Another issue is that until few years ago, there was not available a selective NBC inhibitor. Fortunately in 2008 the group of Dr. Vaughan-Jones presented and characterized a novel and selective NBC inhibitor [7], that has been successfully used to demonstrate the importance of total NBC activity in the control of cardiac pH_i_ [8].

At present it is known that NBC is responsible for 40-50% of total acid extrusion in cardiac myocytes [9,10]. Moreover, although at acidic pH_i_ (near to 6.8) the relative importance of NBC is only of 30% against the 70% of NHE [8,11,12], both transporters are equally operative at pH_i_ closed to basal [8,13,14].

Interestingly, it has been demonstrated that NBC increase the [Na^+^]_i_, being responsible for 30% of this increase at pH_i_ 6.8 [12]. The increase in [Na^+^]_i_ stimulates the reverse mode of Na^+^/Ca^2+^ exchanger (NCX), leading to an increase in [Ca^2+^]_i_ [15-17]. This process is involved in Ang II and Endothelin-1 (ET-1) -induced positive inotropic effects [17,18] and cardiac hypertrophy [19,20]. Furthermore, this phenomenon might be involved in NBC-induced cardiac pathologies [21].

## ELECTRONEUTRAL (nNBC) AND ELECTROGENIC (eNBC) ISOFORMS OF NBC IN THE HEART

Cardiac NBC was initially described by Lagadic-Gossmann *et al.* as an electroneutral transporter (nNBC), with a stoichiometry of 1 Na^+^/1 HCO_3_^-^[9]. Some years later Dr. Cingolani’s group demonstrated that NBC exhibits an electrogenic behavior (eNBC), with a stoichiometry of 1 Na^+^/2 HCO_3_^-^ [10]. In addition, we have later described and characterized the eNBC current as an anionic bicarbonate and sodium-dependent current which reversed at around -85 mV (I_NBC_) [22,23]. Moreover, the functional diversity of the eNBC in ventricular myocytes from rat, rabbit and guinea pig has been described in detail by Yamamoto *et al*. [24]. 

The controversy around the cardiac NBC stoichiometry has been resolved and it is now accepted that both mechanisms are present in cardiac cells [13,25]. In the heart exist at least two electrogenic isoforms, called NBCe1 (also named NBC1) and NBCe2 (also named NBC4) which are encoded by the SLC4A4 gene [26], and the SLC4A5 gene [27], respectively, and one electroneutral isoform, named NBCn1 (also named NBC3), that is encoded by the SLC4A7 gene [28].

We have described the influence of eNBC in the configuration of the cardiac action potential (CAP) [27]. Using the patch-clamp technique, we have demonstrated that the change of the extracellular solution from a HEPES- (HCO_3_^-^-free solution) to a HCO_3_^-^- containing solution hyperpolarized resting membrane potential (RMP) by 3-5 mV and evoked a 25% CAP shortening, both in rat [22] and cat [23] ventricular myocytes. Reciprocally, it has been shown that eNBC increased pH_i_ in response to the change in RMP induced by hiperkalemic extracellular solutions [10,24,29] or after increasing the heart rate [30].

While the roles of NBCe1 and NBCn1 have been well established, the true relevance of the NBCe2 is still unresolved. Although it was initially reported to be present in the heart, the existence of NBCe2 in the cardiomyocytes was recently challenged [31,25]. Moreover the NBCe1 seems to be the only active electrogenic mechanism in normal cat and rat ventricular myocytes [25,32]. We have also recently reported that this NBC isoform is physically and functionally coupled to the carbonic anhidrase in rat ventricular myocytes [33]. 

A novel contribution to the knowledge about NBC was presented by us last year [25]: We have produced and characterized two different and selective functional antibodies against the extracellular loops of NBCe1, that were called a-L3 and a-L4, which recognized the extracellular loop 3 and loop 4, respectively. The pre-incubation of the myocytes with a-L3 canceled NBCe1 function, which allowed us to demonstrate that this isoform was the main, if not the only, electrogenic active isoform in normal cardiac myocytes. On the other hand, the pre-incubation with a-L4 improved the NBCe1 activity [25]. Inhibitory antibodies of NBCe1 have been previously used to investigate the implication of this isoform in the contractile dysfunction induced by ischemia-reperfusion [34]. However, a direct activator of NBCe1 has never been used before our work. Nevertheless, besides that NBCe1 seems to be the only active electrogenic isoform in cardiac myocytes under physiological conditions, it still unknown the relevance of each NBC isoform during the development and progress of cardiac pathology. Thus, we proposed these antibodies as new pharmacological tools that will allow us to investigate the participation of NBCe1 in isolation in cardiac pathophysiology. 

## SYSTEMIC AND LOCAL ANG II IN THE HEART

Ang II is an important hormone that regulates the excitation and contraction in the heart. Ang II is an octapeptide that was classically known to be synthesized from Ang I by the angiotensin-converting enzyme (ACE) present in the endothelial vessels in response to increases levels of Aldosterone (Ald), conforming the endocrine system known as renin- angiotensin- aldosterone-system (RAAS). Moreover, at present it is well recognized that Ang II is produced and secreted in several tissues, including the heart [35]. Dr. Sadoshima’s group has shown for the first time that Ang II exerts autocrine and paracrine effects when it is secreted from intracellular vacuoles in response to myocyte stretching, leading to cardiac hypertrophy [36,37]. Furthermore, Dr. Cingolani’s group have deeply investigated the presence of this autocrine pathway in the slow force response (SFR) to myocardial stretch, proposing the NHE stimulation as the final effect triggered by the endogenous Ang II action [38,39]. Furthermore, it was shown that >75% of cardiac Ang II was synthesized locally, and that its source was also in situ-synthesized Ang I [40]. In concordance, it has been demonstrated that Ald synthase exist in the myocyte [41], supporting the existence of a local RAAS [42]. On the other hand, it is important to mention that, under pathological conditions, like post-myocardial infarction and in response to pressure and volume overload, increased cardiac Ang II levels [43-45] and upregulation of AT-1 receptors [46] were reported. 

It is well-known that Ang II effects involve MAP kinases (MAPK) stimulation and reactive oxygen species (ROS) generation [47-50]. In this regard, it has been demonstrated that low concentration of ROS, instead of being deleterious, acts as intracellular molecules that regulate myocyte physiology [50-52]. Moreover, the exposure of cardiac myocytes to extracellular H_2_O_2_ activates the ERK 1/2 kinase and stimulates NHE in a dose and time- dependent manner [53,54]. 

Interestingly, at present it is accepted that many effects initially thought to be produced directly by Ang II, are really induced by ET-1 [19,55], Ald [56,57] and more recently, after the transactivation of the epidermal growth factor receptor (EGFR) [58-61]. Because of the close relationship between Ang II and the regulation of ion membrane transporters, in the last few years the investigation of Ang II-induced NBC modulation gained increasing interest.

## REGULATION OF NBC BY ANG II

It was demonstrated that Ang II stimulates total NBC activity during the recovery of pH_i_ after an intracellular acidosis both in rat [11] and cat [8] adult ventricular myocytes in a ROS- [8] and ERK 1/2- dependent manner [8,11]. Moreover, the recently described phenomenon of “ROS-induce-ROS-release” [62-64] was also involved in Ang II-induced NBC stimulation [8]. In addition, in neonatal rat myocytes, Ang II was reported to simulate NBC activity in a phosphoinositide-independent mechanism after activation of AT-2 receptors [65].

**Fig. (1):**
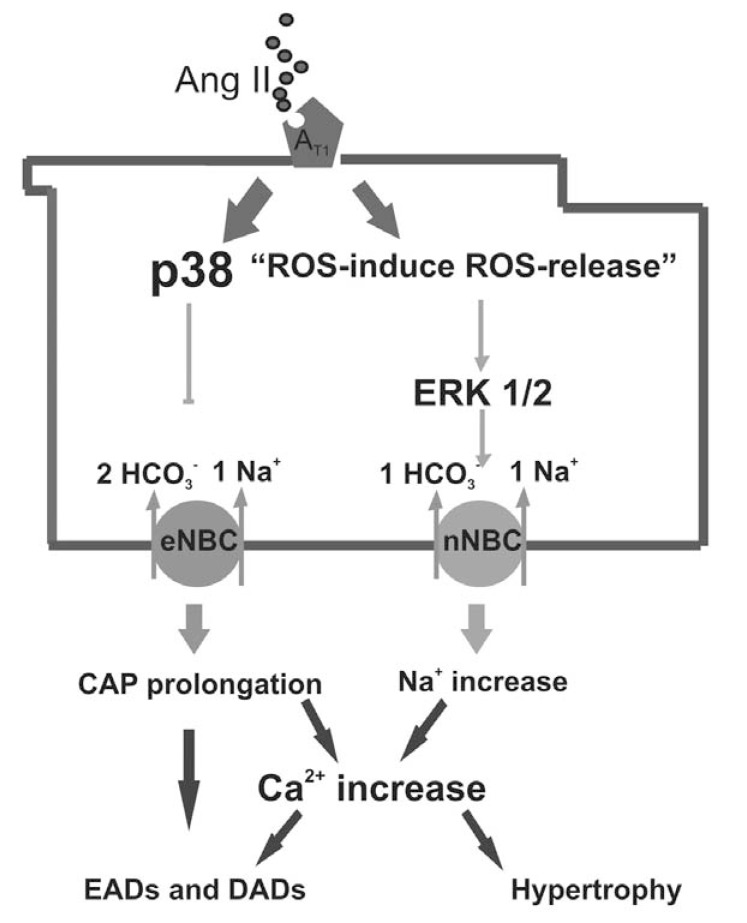
Schematic diagram showing the proposed Ang II- induced opposite effects on NBC isoforms and the possible implications in cardiac pathologies, as hypertrophy and arrhythmias. p38: p38 kinase; ERK 1/2: ERK kinase.

We have recently shown for the first time a differential effect of Ang II on NBC isoforms [29]. We demonstrated that Ang II inhibits eNBC in a p38 kinase-dependent, but ERK 1/2 and ROS-independent manner, whereas activates nNBC via an ERK 1/2 and ROS-dependent mechanism (Fig. **1**). Thus, we suggested that Ang II, binding to the AT-1 receptor, activates the nNBC and inhibits the eNBC through parallel pathways [29]. Since previous studies, in which the effect of the hormone on both isoforms was not discriminated, have shown that Ang II stimulates total NBC activity [8,11], it might be possible to speculate that Ang II-induced stimulation of nNBC is able to overrule the inhibition of eNBC [29]. Consistently, when we measured total NBC activity during the recovery from acidosis, we found a stimulatory effect of Ang II which was further enhanced when p38-kinase was blocked, demonstrating that Ang II-induced inhibiton of eNBC was only partly compensating the excitatory effect of the hormone on nNBC [29]. Furthermore, in the presence of NHE inhibition with HOE642, Ang II also significantly increased resting pH_i_, again likely due to nNBC stimulation overcoming eNBC inhibition [29].

Although we presented the first evidence for the Ang II-induced cardiac eNBC inhibition, this effect was in agreement with several studies that demonstrated a biphasic regulation of NBCe1 by Ang II in renal tubules: low concentrations (picomolar to nanomolar) stimulated NBCe1 activity whereas higher concentrations (nanomolar to micromolar) inhibited it in an arachidonic acid-dependent way [66,67]. Consistently, it has been previously reported that p38 kinase is related with the arachidonic acid pathway and its metabolites in several tissues [68-71]. Further investigations are needed to evaluate the participation of arachidonic acid in Ang II-induced cardiac eNBC regulation. Moreover, it would be interesting to investigate the effect of low concentrations of Ang II in cardiac NBC activity.

## INVOLVEMENT OF NBC IN CARDIAC PATHOLOGY

Although little is yet known about the implication of NBC in cardiac pathologies, in the last years there was a considerable increase in the knowledge about this issue, which suggested the involvement of NBC in several heart diseases, such as myocardial ischemia [34,72-77], infarction [78] and cardiac hypertrophy [31,79]. Interestingly, Ang II is also involved in these pathologies. 

### a) Ischemic Disease and Myocardial Infarction 

Myocardial infarction is a major cause of death and disability worldwide. It is mainly developed during an unstable period of a coronary atherosclerosis disease. The term myocardial infarction reflects the existence of cardiomyocyte death caused by prolonged ischemia, which is the result of a perfusion imbalance between supply and demand. 

Previous studies have demonstrated that cardiac NBC is activated during ischemia-reperfusion [72-77]. Moreover, Khandoudi and coauthors [34] have demonstrated that selective inhibition of NBCe1 during reperfusion after ischemia significantly improved contractile recovery, indicating that this transporter contributes to the characteristic intracellular Na^+^ and Ca^2+^ overload produced by this pathology. These authors also showed that NBCe1 is over-expressed in human heart failure [34].

Experimentally, it was demonstrated that local myocardial infarction (MI) leads to an increase in both, mARN level and NBC protein expression and, as a consequence, NBC activity was enhanced [78]. Chronic treatment with blockers of the Ang II signaling, either with ACE inhibitor or AT-1 receptor antagonists, effectively reduced mRNA and protein NBC upregulation and transport activity [78], demonstrating a close relationship between NBC, Ang II and myocardial infarction.

### b) Cardiac Hypertrophy 

Cardiac hypertrophy is a response of the heart to a variety of extrinsic and intrinsic stimuli, some of which finally lead to a maladaptative state. On the basis of the external stimuli and ongoing molecular changes, cardiac hypertrophy is divided in two types; the physiological cardiac hypertrophy, mostly seen in athlete’s heart and the pathological cardiac hypertrophy induced by mechanical stress, due to pressure overload or volume overload. In the physiological cardiac hypertrophy the increase of the cardiac muscle reduces ventricular wall stress and compensates for the increased hemodynamic demand, improving heart contractility. It is generally associated with increased cardiac mass without collagen deposition. Opposite, the pathological cardiac hypertrophy is characterized by the fibrosis and induction of fetal gene expression, which lead to a reduction in cardiac output and enhancement of the risk of sudden death, arrhythmia and heart failure [80,81].

Yamamoto *et al.* have demonstrated that NBCe1 and NBCn1 were over-expressed in ventricular myocytes isolated from hypertrophied rat hearts subjected to non-ischemic pressure overload [31]. Moreover, these changes are prevented by Losartan [31]. When these authors evaluated the function of both isoforms in the hypertrophied hearts, they could not find a clear upregulation of NBCe1 [31]. Consistently, we have recently shown preliminary data suggesting that, although NBCe1 is also over-expressed in hypertrophied hearts of spontaneous hypertensive rats (SHR), its activity is impaired [79]. It is possible that Ang II induced the NBCe1 internalization, explaining the discordance between the protein expression and the transport activity. In agreement, Ang II-induced NBCe1 internalization was described in Xenopus oocytes transfected with this NBC isoform [82]. Nevertheless, it is important to mention that it could not be determined yet if the changes on NBC were the cause or the consequence of the development of cardiac hypertrophy. Additional studies are required to fully resolve this important issue. 

## ROLE OF NBC-INDUCED [NA^+^]_i_ AND [CA^2+^]_i_ OVERLOAD: POTENTIAL IMPLICATIONS IN CARDIAC HYPERTROPHY

It is well-known that increased [Ca^2+^]_i_ activates hypertrophic pathways, such as the one of calcineurin [83,84]. Ca^2+^ regulation is closely linked to [Na^+^]_i_ because one of the routes for Ca^2+^ influx into the myocytes is via the reverse mode of NCX. When [Na^+^]_i_ increases, NCX is shifted to less forward mode activity (Ca^2+^- efflux) and/or to reverse operation mode, leading to [Ca^2+^]_i_ overload [85-87].

In animal models of hypertrophy, as well as in human heart failure, it has been demonstrated an increase in [Na^+^]_i_ and [Ca^2+^]_i_ [88-90]. Furthermore, it was shown that chronic inhibition of NHE, which attenuates the [Na^+^]_i_ overload, prevented or reverted cardiac hypertrophy [91-94]. On the other hand, the over-expression of NHE induced cardiac hypertrophy [95].

As it was demonstrated that NBC is responsible for 30% of Na^+^ influx into the myocyte at pH_i _6.8 [12], it may be also important in the development of cardiac hypertrophy. In this regard and as commented above, it has been shown that nNBC function is up-regulated in cardiac hypertrophy [31], while eNBC transport seems to be impaired [79]. Taking into account the stoichiometry of both NBC isoforms, which could lead to the consideration of eNBC as a “Na^+^- sparing” bicarbonate transporter, it is feasible to anticipate that this remodeling in NBC isoforms function in the hypertrophied hearts would lead to more deleterious effects on [Na^+^]_i_ and [Ca^2+^]_i_ overload. 

## ROLE OF NBC- INDUCED [Na^+^]_i_ AND [Ca^2+^]_i_ OVERLOAD: POTENTIAL IMPLICATION IN DELAYED AFTER DEPOLARIZATIONS (DADs)

It has been shown that either the inhibition of the Na^+^/K^+ ^ATPase [96,97] or the NHE stimulation [98] generate [Na^+^]_i_ overload and cardiac arrhythmias. The proposed mechanism is the following: [Na^+^]_i_ overload reduces Ca^2+^ extrusion and/or increases Ca^2+^ influx through the NCX. The increase in [Ca^2+^]_i_ enhance the sarcoplasmic reticulum (SR) calcium load, exceeding the ryanodine receptor channel (RyR) threshold necessary to be opened and finally leading to spontaneous diastolic calcium release. The transient increase in citosolic Ca^2+^ (waves) activates an inward (depolarizing) current (I_ti_), mediated by the forward mode of NCX [99,100]. I_ti _is responsible for the generation of DADs which, when are sufficiently large to achieve the threshold, generate spontaneous CAP, leading to triggered activity [101]. 

As NBC activity promotes the increase in [Na^+^]_i_ [12], it is also possible to speculate that Ang II and ROS-induced NBC stimulation [8] might be implicated in DADs generation (Fig. **1**). According to this, it was demonstrated that Ang II induces DADs in a ROS-dependent manner [102].

However, it is important to note that increased SR Ca^2+^ load is not sufficient to promote diastolic spontaneous SR Ca^2+^ release [97], and that also a functional or structural alteration in RyR is needed to induce DADs [103-107]. In addition, it was recently demonstrated that ROS can directly oxidise the RyR, making it leaky [108-111]. Interestingly, the inverse conclusion seems to be also true: just an impaired RyR is not sufficient to induce Ca^2+^ release, since a parallel increase in SR Ca^2+^ load is also required [112].

## ROLE OF eNBC INHIBITION-INDUCED CAP PROLONGATION: POTENTIAL IMPLICATION IN EARLY AFTER DEPOLARIZATIONS (EADs)

Classically, Ang II is known to modulate the properties of ion channels leading to CAP prolongation ^[113-116]^. It has been reported that Ang II both inhibits repolarizing currents as I_K1_, I_Kr_ and I_to_ [114,116-118] and stimulates depolarizing currents as I_CaL _[119,120]. Moreover, we have recently demonstrated that Ang II abrogated the eNBC-induced CAP shortening, likely due to the inhibition of the repolarizing current generated by the transporter [29].

In this regard, it has been shown that CAP prolongation enhances the occurrence of EADs, due to the recovery from the inactivation and the reactivation of voltage-dependent L-type Ca^2+^ channels [102,121,122] and the impairment of sodium current [102,122]. In addition, Ang II was shown to increase the occurrence of EADs in a ROS and CaMKII-dependent manner [102]. Furthermore, it was shown that chronic inhibition of NHE could reverse the ionic (sodium overload and disturbance in calcium management) and electrical (CAP prolongation) cellular remodeling during heart failure, and reduce arrhythmic events [98].

The relationship between NBC and EADs is currently unknown, but keeping in mind the relevance of eNBC for CAP configuration [29], and the regulation of [Na^+^]_i_ by total NBC activity [31], it might be also possible that Ang II-induced eNBC inhibition and nNBC stimulation participate in the generation of EAD, secondary to CAP prolongation and [Na^+^]_i_ overload, respectively (Fig. **1**). 

Importantly, there is a close relationship between CAP prolongation and hypertrophy. Prolongation of CAP is consistently observed in several experimental models of cardiac hypertrophy and failure [123]. It is also known that this can lead to QT prolongation in the electrocardiogram, that in turn promotes arrhytmogenic events [98,124]. Interestingly, Lebeche *et al.* have reported that CAP prolongation promotes an increase in [Ca^2+^]_i_, which activate a hypertrophic signaling pathway, that might be a cause and not a consequence of cardiac hypertrophy [125]. 

## eNBC ACTIVITY: BENEFICIAL OR DETRIMENTAL? 

**Fig. (2):**
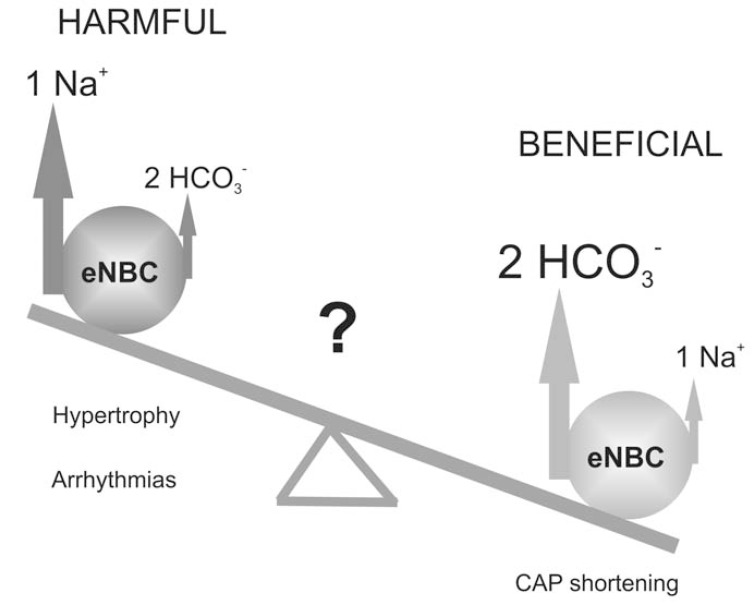
Schematic representation of eNBC activity showing the balance between its beneficial repolarizing current (*right*) and the consequences of its Na^+^-loading effect (*left*) in cardiac pathologies.

The double participation of eNBC in cardiac physiology makes it difficult to call as a “beneficial” or “detrimental” mechanism (Fig. **2**). Since eNBC is a cellular “Na^+^- loading” mechanism, it might contribute to [Na^+^]_i_ and [Ca^2+^]_i_ overload and arrhythmias generation [21,73]. In concordance, Khandoudi *et al.* reported that blockade of rat cardiac eNBC during reperfusion results in cardioprotection [34]. Thus, we can speculate that the Ang II-induced eNBC inhibition would carry beneficial effects under this pathological state. Similar paradigmatic speculations about the effect of Ang II on eNBC could be made with cardiac hypertrophy and heart failure, which are cardiac diseases associated to elevated Ang II concentration [40,126]. 

However, in cardiac hypertrophy or heart failure, the potential attenuation of [Na^+^]_i_ overload that might be produced by eNBC inhibition [31] could be overruled by the deleterious effects that might be carried by the prolongation of CAP also induced by the blockade of eNBC [29]. Moreover, Camilión de Hurtado *et al.* have demonstrated that a rate-dependent decrease in pH_i_, probably due to the increased anaerobic glycolisis, was markedly reduced in the presence of CO_2_/bicarbonate in comparison to a free-bicarbonate solution [30]. In this work, the authors proposed that eNBC activity, which leads to HCO_3_^-^ influx, substantially increases the cell ability to recover from enhanced proton production [30]. 

## FINAL CONCLUSION

The purpose of this review was to focus the attention on the cardiac NBC and specially consider its regulation by Ang II and the implications of this modulation, either in physiology or in the development of cardiac diseases. Classically, the NBC is known as an alkalinizing mechanism. However, it is important to keep in mind that this is not its only function, but it also controls [Na^+^]_i_, and indirectly [Ca^2+^]_i_ through the NCX activity and SR behavior. Moreover, eNBC modulates the shape and the duration of the CAP, adding to this isoform the important role of contributing to cellular electrophysiology.

We consider of significant relevance the fact that a hormone as Ang II, which has a central role in cardiac pathophysiology, regulates NBC activity. Moreover, this peptide exerts an opposite effect on each NBC isoform due to the activation of two different and parallel pathways. The inhibitory effect that Ang II exerts on eNBC, via the activation of the p38-kinase, seems to be more relevant for CAP duration than for pH_i_ regulation. On the opposite, the stimulatory effect of this hormone on nNBC, dependent on ROS production and ERK 1/2- activation, overrule the negative effect on eNBC, leading to an increase in pH_i_, [Na^+^]_i_, and [Ca^2+^]_i_ that could be important to explain, at least in part, the hypertrophic effects of Ang II signaling. 

The knowledge of the singular regulation of each NBC isoform should be the base for following investigations. The use of specific inhibitors of the ERK 1/2 or p38- kinases pathways and the employment of functional antibodies as new pharmacological tools, will allow the study of the differential implication of eNBC and nNBC in cardiac pathologies. As an example of clinical relevance, it is feasible to suggest that the stimulatory antibody (a-L4) against eNBC, which would induce CAP shortening, could be useful to investigate the potential protective effect of eNBC activation during the development of cardiac hypertrophy or the damage during reperfusion after ischemia. Nevertheless, besides the fact that the amount of pharmacological tools has been growing up, we are still in debt, and the more precise knowledge about development of cardiac diseases that we can elucidate, the more close we will be to find the specific treatment for them.
